# Sociodemographic Indicators of Child and Adolescent Mortality in Finland—A Nationwide Study of 310 Municipalities Covering Over 5,000,000 Inhabitants

**DOI:** 10.3389/fpubh.2021.678293

**Published:** 2021-10-13

**Authors:** Petteri Oura, Antti Sajantila

**Affiliations:** ^1^Department of Forensic Medicine, University of Helsinki, Helsinki, Finland; ^2^Forensic Medicine Unit, Finnish Institute for Health and Welfare, Helsinki, Finland

**Keywords:** children, adolescents, mortality, death, sociodemographic, epidemiology, Finland

## Abstract

**Background:** The reduction of child and adolescent deaths (defined as decedents aged 0–19 years) remains a crucial public health priority also in high-income countries such as Finland. There is evidence of a relationship between socioeconomic gradients and child mortality, but the association is considered complex and relatively poorly understood. Exploiting a Finnish dataset with nationwide coverage, the present study aimed to shed light on the sociodemographic predictors of child and adolescent mortality at the municipality level.

**Methods:** A public database of Statistics Finland was queried for municipality-level data on sociodemographic traits and child and adolescent deaths in Finland during the years 2011–2018. The sociodemographic indicators included total population size, child and adolescent population size, sex distribution, mean age, education, unemployment, median income, population density, rurality, percentage of individuals living in their birth municipality, household size, overcrowded households, foreign language speakers, divorce rate, car ownership rate, and crime rate. The sociodemographic indicators were modeled against child and adolescent mortality by means of generalized estimating equations.

**Results:** A total of 2,371 child and adolescent deaths occurred during the 8-year study period, yielding an average annual mortality rate of 26.7 per 100,000 individuals. Despite a fluctuating trend, the average annual decline in child and adolescent deaths was estimated to be 3% (95% confidence interval 1–5%). Of the sociodemographic indicators, population density was associated with higher child and adolescent mortality (rate ratio 1.03, 95% confidence interval 1.01–1.06), whereas the percentage of foreign language speakers was associated with lower child and adolescent mortality (0.96, 0.93–0.99).

**Conclusion:** Densely populated areas should be the primary focus of efforts to reduce child and adolescent mortality. Of note is also the apparently protective effect of foreign language speakers for premature mortality. Future studies are welcomed to scrutinize the mediating pathways and individual-level factors behind the associations detected in this study.

## Introduction

Child well-being is a key public health priority (1), and child mortality is commonly used as an economic and social welfare indicator (2, 3). Although the deaths of children and adolescents [defined as individuals aged 0–19 years (4)] have fortunately become rare in high-income countries (5), these deaths are far from insubstantial and often preventable (5, 6). Endeavors to further reduce child and adolescent mortality should be grounded upon a thorough understanding of the underlying factors that, in turn, could be exploited in the planning of preventive measures and resource allocation. The reduction of child and adolescent mortality remains a crucial public health priority also in high-income countries (7, 8).

The social determinants of health, as defined by the World Health Organization, comprise a wide range of factors that constitute the circumstances one lives in (9). Notably, the list includes common sociodemographic traits such as education level, employment rate, income level, neighborhood characteristics and crime rate. While the association between social inequality and child mortality is widely prevalent in low- and middle-income countries (10), a similar relationship seems to persist also in high-income countries such as Canada (11), the USA (12), and England (13).

Studies from high-income countries have previously reported several sociodemographic indicators of child and adolescent mortality. In a study from Quebec, Canada with data from the years 1990–2005, an areal socioeconomic deprivation score (comprised of employment, low education, and income) was associated with pediatric mortality in several age groups (11). Similarly, a study from the US with data from the years 1969 to 2000 showed that a county-level deprivation index (composite of 17 indicators, including educational, occupational, economic, and housing-related factors) was associated with child mortality (12). A study comprising two birth cohorts with data from 2003 to 2013 found a clear association between lower socioeconomic status and higher child mortality in Sweden (socioeconomic status proxied by income) and England (socioeconomic status proxied by a composite score of income, employment, disability, education, housing, living environment and crime) (13). However, the association between socioeconomic indicators and child mortality is still considered complex and relatively poorly understood (8), warranting further emphasis on the subject. In order to disentangle the effects of sociodemographic traits from each other, a comprehensive approach to the indicator variables and their intercorrelations is essential.

In Finland, 0.5% of all decedents are aged 0–19 years (14). Exploiting a nationwide dataset from Finland, the present study aimed to elucidate the sociodemographic predictors of child and adolescent mortality at the municipality level. The present dataset included a wide range of sociodemographic indicators which were modeled against child and adolescent mortality by means of generalized estimating equations (GEE). The results of this study were expected to provide municipality-level tools for the identification of geographical areas and/or population groups that have higher child and adolescent mortality.

## Materials and Methods

### Dataset

We used publicly available municipality-level data on sociodemographic indicators and child and adolescent mortality in Finland during the years 2011–2018. The data package was queried between October 2020 and January 2021 from StatFin portal (14), an umbrella database for official national statistics of Finland, which are collected and published by Statistics Finland (15). All data were collected at the municipality level according to Finland's most recent regional division into 310 municipalities. Data collection was restricted to the years 2011–2018 as these were the years when all the variables of interest were available.

### Child and Adolescent Mortality

In Finland, all deaths are notified to the population register at the beginning of the cause-of-death investigation (16). Once the investigation is complete, a death certificate is issued and sent to forensic pathologists at the Finnish Institute for Health and Welfare for an independent review. Accepted certificates are forwarded to Statistics Finland. The Finnish death certification practices have high annual coverage and are thus well-suited for mortality statistics studies (17).

For the present study, “children and adolescents” were defined as individuals aged 0–19 years (4). The StatFin registry was queried for the total number of child and adolescent deaths and the total number of children and adolescents residing in the municipalities at the end of each year. Crude mortality rate was calculated as the number of decedents relative to the child and adolescent population of the municipality.

### Sociodemographic Indicators

Detailed descriptions of the sociodemographic variables have been provided in Supplementary Table 1 and a previous publication (18). In brief, StatFin is a database for official national statistics collected from various authorities. The target population were Finnish citizens and foreigners who were legally residing in Finland for at least 1 year.

Data on population structure [i.e., total population size at the end of the year, child and adolescent population size, percentage of females, mean age, percentage of foreign language speakers (defined as individuals whose primary language is not Finnish, Swedish or Sámi), and percentage of individuals living in their birth municipality] were provided by the Finnish population register maintained by the Digital and Population Data Services Agency. Data with a geographical link (i.e., population density per km^2^ and percentage of individuals living in rural areas) were constructed on the basis of the population register, National Land Survey of Finland, and Finnish Environment Institute.

Educational data [i.e., percentage of individuals with low education (defined as compulsory primary education with no further studies)] originated from various databases that are mainly updated by educational institutions. Employment data [i.e., unemployment rate (defined as unemployed relative to labor force)] were collected from various authorities, including Finnish Tax Administration, Social Insurance Institution of Finland, employment registers, and student and conscript registers. Income data [i.e., median income (defined as annual gross income)] were obtained from the records of Finnish Tax Administration.

Data on housing [i.e., average number of individuals per household unit and percentage of overcrowded household units (defined as units where resident count exceeds the room count excluding kitchen)] were provided by the population register together with Finnish Tax Administration and municipal building inspection authorities. Divorces were reported by courts of law to the population register. Car registrations were collected from the traffic affairs register of Finnish Transport and Communications Agency. Data on all reported crimes were obtained from the Ministry of Interior's police information system.

### Statistical Methods

General characteristics of the municipalities were represented as means with standard deviations or medians with interquartile ranges, depending on the distribution of the data. The general characteristics were calculated for the full sample as well as for rough population size tertiles (<4,000, 4,000–9,999, ≥10,000 inhabitants). One-way analysis of variance and Kruskal-Wallis test were used to analyze the differences between the tertiles.

As the main approach, GEE models were used to analyze the association between sociodemographic indicators and child and adolescent mortality (19). In GEE, the negative binomial distribution was used to model the data with the log link function. The number of child and adolescent deaths was used as the outcome, the total child and adolescent population size as the offset variable, the total population size as the scale weight variable, and the sociodemographic variables as predictors. Annual data were considered to be nested within municipalities. The potential intercorrelations between variables within the dataset were accounted for by means of the “exchangeable” working correlation matrix. The scales of the predictor variables were standardized (i.e., mean and standard deviation set to 0 and 1, respectively) in order to unify the interpretation of regression coefficients. Univariate models were run as the initial step, after which the final full multivariable model was run, incorporating all predictors in the same model. There were no missing values. The exponentiated regression coefficients (i.e., rate ratios, RRs), 95% confidence intervals (CIs) and *P*-values were documented from the GEE output.

The statistical analysis was performed using SPSS Statistics (IBM, Armonk, NY, USA) version 26. The threshold for statistical significance was set at *P* = 0.05.

### Ethical Considerations and Study Permission

Ethical approvals were not required as the dataset was already public, retrospective, register-based, and did not involve individual-level data. Reuse of the StatFin material is permitted in accordance with the CC BY 4.0 license (20).

## Results

The analysis was based on 310 municipalities with a median population size of 6,191 (interquartile range 2,901–14,689). The general sociodemographic characteristics of the municipalities are presented in Table 1. On average, 49.5% of the population were women, 2.0% were foreign language speakers, 34.9% only had primary education, and 11.9% were unemployed. A total of 2,371 child and adolescent deaths occurred, yielding an average annual mortality rate of 26.7 per 100,000 individuals. During the study period 2011–2018, the annual number of child and adolescent deaths fluctuated between 247 and 358 cases (Supplementary Table 2; Supplementary Figure 1). On average, both the number of deaths and mortality rate showed a decline of 3% per year (95% CI 1–5%) (Supplementary Figure 1; Table 2).

**Table 1:** Sociodemographic characteristics of the municipalities.

**Indicator**	**All municipalities**	**Municipalities stratified by population size**			
	**(*n* = 310)**	**<4,000 (*n* = 108)**	**4,000–9,999 (*n* = 99)**	**≥10,000 (*n* = 103)**	***P*-value**
**Child and adolescent mortality**					
Total number of deaths	2,371	106 (4.5%)	328 (13.8%)	1,937 (81.7%)	–
Mortality rate (per 100,000 individuals)	26.7	26.5	28.3	25.4	–
**Population structure**					
Child and adolescent population size^*^	1,332 (569–3,287)	438 (282–588)	1,367 (1,088–1,831)	5,210 (3,370–10,505)	**<0.001**
Total population size^*^	6,191 (2,901–14,689)	2,287 (1,531–3,057)	6,565 (5,121–8,184)	21,436 (15,209–40,855)	**<0.001**
Females (%)	49.5 (1.3)	48.6 (1.4)	49.5 (0.9)	50.5 (0.8)	**<0.001**
Mean age (years)	45.4 (4.3)	47.8 (3.9)	45.2 (4.3)	42.8 (3.2)	**<0.001**
**Education and income**					
Low education (%)	34.9 (5.7)	38.8 (4.7)	34.8 (4.6)	30.6 (4.4)	**<0.001**
Unemployment (%)	11.9 (4.4)	11.9 (5.0)	12.0 (4.4)	11.8 (3.8)	0.882
Median annual income (eur)	21,668 (3,375)	19,946 (2,914)	21,596 (3,270)	23,615 (2,868)	**<0.001**
**Location and housing**					
Population density (per km^2^)^*^	10.7 (5.2–25.1)	5.2 (3.4–9.0)	10.0 (5.7–15.8)	33.3 (19.1–84.6)	**<0.001**
Individuals living in rural area (%)^*^	98.8 (30.3–99.3)	99.1 (98.7–99.4)	98.9 (91.7–99.3)	19.1 (4.2–97.6)	**<0.001**
Individuals living in municipality of birth (%)	50.5 (12.1)	53.7 (9.2)	51.2 (13.2)	46.1 (12.6)	**<0.001**
Individuals per household unit	2.1 (0.2)	2.1 (0.2)	2.2 (0.3)	2.1 (0.2)	**<0.001**
Overcrowded household units (%)	8.8 (2.1)	8.9 (2.3)	9.0 (2.3)	8.3 (1.5)	**<0.001**
**Other indicators**					
Foreign language speakers (%)^*^	2.0 (1.2–3.3)	1.6 (1.0–3.0)	1.7 (1.2–2.5)	2.7 (1.9–4.2)	**<0.001**
Divorce rate (per 1,000 individuals)	2.0 (0.9)	1.7 (1.1)	1.9 (0.7)	2.4 (0.5)	**<0.001**
Car ownership rate (per individual)	0.66 (0.10)	0.72 (0.12)	0.66 (0.06)	0.61 (0.07)	**<0.001**
Crime rate (per individual)^*^	0.11 (0.08–0.16)	0.08 (0.06–0.12)	0.12 (0.09–0.19)	0.13 (0.11–0.17)	**<** **0.001**

*Values are means with standard deviations unless otherwise indicated*.

* *Values are medians with interquartile ranges. Bold denotes statistical significance*.

**Table 2:** Sociodemographic indicators of child and adolescent mortality.

**Indicator**	**Univariate models**			**Full multivariable model**		
	**RR**	**95% CI**	***P*-value**	**RR**	**95% CI**	***P*-value**
Year (average annual change)	**0.97**	**0.95; 0.98**	**<0.001**	**0.97**	**0.95; 0.99**	**<0.001**
**Population structure**
Females (%)	1.01	0.96; 1.05	0.774	0.91	0.83; 1.00	0.054
Mean age (years)	**1.14**	**1.06; 1.23**	**<0.001**	0.92	0.81; 1.04	0.185
**Education and income**
Low education (%)	1.06	0.99; 1.13	0.102	1.07	0.97; 1.19	0.178
Unemployment (%)	**1.07**	**1.00; 1.14**	**0.039**	1.00	0.89; 1.11	0.938
Median annual income (eur)	**0.91**	**0.86; 0.96**	**<0.001**	0.95	0.83; 1.08	0.429
**Location and housing**
Population density (per km^2^)	1.00	0.99; 1.01	0.799	**1.03**	**1.01; 1.06**	**0.007**
Individuals living in rural area (%)	**1.09**	**1.04; 1.14**	**<0.001**	1.05	0.96; 1.16	0.280
Individuals living in municipality of birth (%)	**1.11**	**1.06; 1.16**	**<0.001**	1.00	0.93; 1.07	0.978
Individuals per household unit	**0.92**	**0.88; 0.97**	**0.002**	0.91	0.79; 1.05	0.179
Overcrowded household units (%)	**0.93**	**0.87; 0.99**	**0.028**	0.94	0.84; 1.05	0.244
**Other indicators**
Foreign language speakers (%)	0.98	0.95; 1.01	0.172	**0.96**	**0.93; 0.99**	**0.019**
Divorce rate (per 1,000 individuals)	0.94	0.87; 1.01	0.102	1.02	0.92; 1.13	0.670
Car ownership rate (per individual)	1.02	0.98; 1.08	0.329	0.99	0.89; 1.09	0.789
Crime rate (per individual)	1.04	0.96; 1.13	0.326	1.01	0.94; 1.09	0.785

*Effect sizes of each predictor are interpreted in standard deviation units. Intermediate models are presented in Supplementary Table 3. Bold denotes statistical significance*.

*CI, Confidence interval; RR, Rate ratio*.

Univariate and full multivariable GEE models for the association between sociodemographic indicators and child and adolescent mortality are shown in Table 2; intermediate models are presented in Supplementary Table 3. Effect sizes (i.e., RRs for mortality) are presented relative to one standard deviation change in the predictor variable. In the preliminary univariate models, higher mortality was associated with population mean age, unemployment, rurality, and higher percentage of individuals living in their birth municipality. Correspondingly, lower mortality was associated with higher income, family size, and higher percentage of overcrowded households. In the full multivariable model, where the internal dependencies within the dataset were controlled for, population density was associated with higher child and adolescent mortality (rate ratio 1.03, 95% confidence interval 1.01–1.06), and the percentage of foreign language speakers was associated with lower child and adolescent mortality (0.96, 0.93–0.99).

## Discussion

Using a comprehensive dataset on 310 Finnish municipalities from the period 2011–2018, this study aimed to explore the association between sociodemographic indicators and child and adolescent mortality at the municipality level. Despite a fluctuating trend, the present analysis estimated child and adolescent deaths to have declined at an average rate of 3% per year over the study period. After accounting for the intercorrelations between the sociodemographic variables, child and adolescent mortality was associated with higher population density and lower percentage of foreign language speakers. The present findings are expected to advance the identification of geographical areas and population groups with higher child and adolescent mortality.

Child and adolescent mortality has undergone a significant decrease in Finland during the previous century (21). Although the number of annual deaths fluctuated over the study period, the positive decline seems to have continued also in the 2010s, thanks to investments in pediatric healthcare, child welfare and traffic safety, among other advances. It is, however, worth acknowledging that a total of 2,371 deaths still occurred during the study period. As the majority child and adolescent deaths are generally considered preventable (5), a need clearly remains for further research and actions enhancing the preventive process. Correspondingly, the differences between univariate and multivariable associations of the sociodemographic indicators with child and adolescent mortality underline the intercorrelated nature of the sociodemographic indicators behind mortality (8). In the full multivariable model, child and adolescent mortality was independently associated with only two sociodemographic indices, namely population density (positive association) and percentage of foreign language speakers (inverse association). However, it is worth acknowledging that the effect sizes of all predictors tended to be relatively small, indicating that the sociodemographic indices only have mild predictive power over child and adolescent mortality.

The positive association between population density and mortality has been previously reported in adult populations from the Netherlands (22), Denmark (23) and Japan (24). A number of socioeconomic indicators such as unemployment, poor housing conditions and crime rate were suggested as potential effect mediators. Quite surprisingly, the present analysis of child and adolescent mortality identified population density as the only socioeconomic or housing-related indicator which had an independent association with the outcome. It is particularly interesting that indices such as income, crime rate, and car ownership rate showed no association with mortality. While the generally low population density in Finland, residual confounding, and municipality-level perspective may explain the differing findings, future studies are encouraged to characterize the factors underlying this discrepancy. We speculate that high population density may also reflect “relative poverty”; even in a country with a low level of income inequality such as Finland (25), our finding would highlight this aspect of poverty upon children's lives. Nonetheless, children and adolescents living in Finnish municipalities with high population density have higher mortality than those living in less densely populated areas, even when total child and adolescent population sizes are accounted for. It would therefore stand to reason to primarily focus efforts to reduce child and adolescent mortality on densely populated areas.

Both the previous evidence of foreign language speakers associating with higher birth rate (18) and the present association between foreign language speakers and lower child and adolescent mortality underline the positive contribution of this population group to the Finnish demography and child well-being. There are also previous reports of lower child mortality among foreign language speakers from Canada (26) and the USA (27). The immigrant background in the majority of foreign language speakers is likely to explain the differences to the general population. In particular, culture-related health behaviors have been suggested as a protective factor in certain areas of health (27). However, foreign language speakers encompass a greatly diverse and heterogeneous group of individuals and families in terms of ethnic backgrounds and cultural traditions, which complicates drawing detailed conclusions from this population group. It may be that municipalities which offer a warm welcome for foreign language speakers and take their convenience into account in public services, also perform better in the prevention of child and adolescent mortality. In a wider sense, we speculate that the finding might reflect the “migrant drive” (28) or “immigrant paradox” (27) that incomers bring to their host society.

The main strength of this study was the nationwide coverage of 310 municipalities and an 8-year study period. The dataset was comprised of official national statistics collected by Statistics Finland from public authorities and registers. Data collections were performed systematically across the nation without regional discrepancies. As the data originate from a public data portal, they are openly available for confirmatory analyses and future research openings. Numerous sociodemographic variables were covered, and their intercorrelations were accounted for in the GEE analysis.

There were also limitations to this study. As we addressed the relationship between sociodemographic traits and child and adolescent mortality only at the municipality level, our results may not be extrapolated to the individual level. There may also be variance in the coverage and quality of register-based data. All-cause mortality was modeled instead of cause- or age-specific scrutiny due to the low number of outcome events. We fully acknowledge that factors such as age group and cause of death may moderate the relationship between sociodemographic variables and mortality, but these could not be addressed in our dataset due to significant floor effects (i.e., most municipalities having zero events, and only a few having more than one). Unfortunately, data on health indicators such as smoking, physical activity or obesity were not available to us. The observational nature of the study and time-series approach prevented conclusions regarding cause-effect-relationships. Although we were able to incorporate a wide range of sociodemographic traits in our multivariable analysis, we cannot fully rule out residual confounding. We hope that future studies will be able to address and compensate the limitations of our study.

In conclusion, this nationwide study of 310 Finnish municipalities aimed to identify sociodemographic indicators of child and adolescent mortality at the municipality level. An average annual decrease of 3% in child and adolescent deaths occurred during the study period. Higher population density was associated with higher mortality, and higher percentage of foreign language speakers with lower mortality. Densely populated areas should be the primary focus of efforts to reduce child and adolescent mortality. Future studies are welcomed to scrutinize the mediating pathways and individual-level factors behind the detected associations.

## Data Availability Statement

Publicly available datasets were analyzed in this study. This data can be found here: https://www.stat.fi/tup/tilastotietokannat/index_en.html (StatFin database, Statistics Finland).

## Ethics Statement

Ethical review and approval was not required for the study on human participants in accordance with the local legislation and institutional requirements. Written informed consent from the participants' legal guardian/next of kin was not required to participate in this study in accordance with the national legislation and the institutional requirements.

## Author Contributions

PO and AS contributed to conception of the study. PO organized the database, performed the statistical analysis, and wrote the first draft of the manuscript. All authors contributed to manuscript revision, read, and approved the submitted version.

## Conflict of Interest

The authors declare that the research was conducted in the absence of any commercial or financial relationships that could be construed as a potential conflict of interest.

## Publisher's Note

All claims expressed in this article are solely those of the authors and do not necessarily represent those of their affiliated organizations, or those of the publisher, the editors and the reviewers. Any product that may be evaluated in this article, or claim that may be made by its manufacturer, is not guaranteed or endorsed by the publisher.
