# Supplementary material for: Sociodemographic Indicators of Child and Adolescent Mortality in Finland—A Nationwide Study of 310 Municipalities Covering Over 5,000,000 Inhabitants
Source: Front Public Health. 2021 Oct 13;9:678293. doi: 10.3389/fpubh.2021.678293 (PMC8548607; doi:10.3389/fpubh.2021.678293)
Supplement: Supplementary file 1 [file Data_Sheet_1.PDF]

Supplementary Table 1. Descriptions of sociodemographic variables.

Sociodemographic indicator	Definition	External link to full variable description	External reference
Total population size	Finnish citizens and foreigners who permanently resided in a municipality on Dec 31; based on data from the population register.	http://www.stat.fi/til/vaerak/meta_en.html http://www.stat.fi/til/vaerak/kas_en.html http://tilastokeskus.fi/meta/kas/kaupunki_maaseu_en.html	Official Statistics of Finland (OSF): Population structure [e-publication]. ISSN=1797-5395. Helsinki: Statistics Finland.
Child and adolescent population size	Residents aged 0—19 years on Dec 31; based on data from the population register.		
Females	Percentage of females; based on data from the population register.		
Mean age	Average age of residents in years; based on data on age on Dec 31 according to the population register.		
Individuals living in municipality of birth	Percentage of residents living in the same municipality where they were born; based on data from the population register.		
Population density	Total number of residents per square kilometer; based on data from the population register, National Land Survey of Finland and Finnish Environment Institute.		
Individuals living in rural area	Percentage of residents living outside urban settlements and peri-urban area; based on data from the population register, National Land Survey of Finland and Finnish Environment Institute.		
Foreign language speakers	Percentage of residents whose primary language is not Finnish, Swedish or Sami; based on data from the population register.		
Low education	Of the population aged ≥15, percentage of residents who have only attained compulsory primary education without secondary or tertiary level qualifications or degrees; based on data from multiple authorities.	http://www.stat.fi/til/vkour/meta_en.html http://www.stat.fi/til/vkour/kas_en.html	Official Statistics of Finland (OSF): Educational structure of population [e-publication]. ISSN=2242-2919. Helsinki: Statistics Finland.
Unemployment	Of the labor force, percentage of unemployed individuals; based on data from multiple authorities.	http://www.stat.fi/til/tyokay/meta_en.html http://www.stat.fi/til/tyokay/kas_en.html	Official Statistics of Finland (OSF): Employment [e-publication]. ISSN=2323-6825. Helsinki: Statistics Finland.
Median annual income	Among all recipients of taxable income within a year, median gross income in EUR; based on data from the Finnish Tax Administration.	http://www.stat.fi/til/tvt/meta_en.html http://www.stat.fi/til/tvt/kas_en.html	Official Statistics of Finland (OSF): Taxable incomes [e-publication]. Helsinki: Statistics Finland.
Individuals per household unit	Total dwelling population (i.e., those who permanently resided in dwellings on Dec 31) relative to the number of households (i.e., a dwelling which is intended for year-round habitation); based on data from the population register, Finnish Tax Administration and municipal building inspection authorities.	http://www.stat.fi/til/asas/meta_en.html http://www.stat.fi/til/asas/kas_en.html	Official Statistics of Finland (OSF): Dwellings and housing conditions [e-publication]. ISSN=1798-6761. Helsinki: Statistics Finland.
Overcrowded household units	Percentage of households in which the number of permanent residents exceeds one person per room (kitchen not included); based on data from the population register, Finnish Tax Administration and municipal building inspection authorities.		
Divorce rate	Number of divorce decrees granted by courts of law relative to total population (given per 1000 individuals); based on data from the population register.	http://www.stat.fi/til/ssaaty/meta_en.html https://www.stat.fi/til/ssaaty/kas_en.html	Official Statistics of Finland (OSF): Changes in marital status [e-publication]. ISSN=1797-643X. Helsinki: Statistics Finland.
Car ownership rate	Number of registered road vehicles (i.e., running on wheels and intended for use on roads) relative to total population; based on data from the Finnish Transport and Communications Agency.	http://www.stat.fi/til/mkan/meta_en.html http://www.stat.fi/til/mkan/kas_en.html	Official Statistics of Finland (OSF): Motor vehicle stock [e-publication]. Helsinki: Statistics Finland.
Crime rate	Total number of reported crimes relative to total population; based on data from the police information system.	http://www.stat.fi/til/rpk/meta_en.html http://www.stat.fi/til/rpk/kas_en.html	Official Statistics of Finland (OSF): Statistics on offences and coercive measures [e-publication]. ISSN=2342-9178. Helsinki: Statistics Finland.

All referred Aug 31, 2021.

Supplementary Table 2. Child and adolescent mortality in Finland over the years 2011—2018.

Year	Total number of deaths	Mortality rate (per 100 000 individuals)
2011	358	29.4
2012	356	29.4
2013	277	22.9
2014	298	24.8
2015	247	20.6
2016	263	22.0
2017	294	24.8
2018	278	23.6

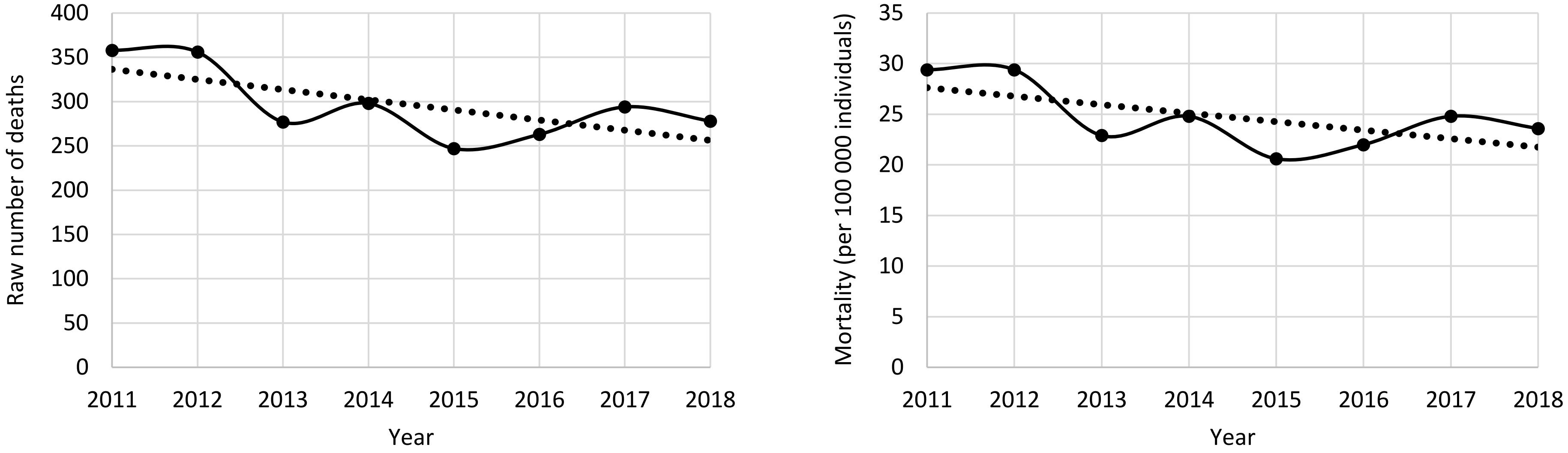

Supplementary Figure 1. Trends in child and adolescent deaths (left) and mortality (right) in Finland during the years 2011—2018. Dashed lines are used to indicate a linear trend.

Supplementary Table 3. Univariate, intermediate, and full multivariable models for the association between sociodemographic indicators and child and adolescent mortality.

Indicator	Univariate models		Intermediate model #1		Intermediate model #2		Intermediate model #3		Intermediate model #4		Full multivariable model	
	RR (95% CI)	P	RR (95% CI)	P	RR (95% CI)	P	RR (95% CI)	P	RR (95% CI)	P	RR (95% CI)	P
Year (average annual change)	**0.97 (0.95; 0.98)**	**<0.001**	**0.97 (0.95; 0.98)**	**<0.001**	**0.97 (0.95; 0.99)**	**<0.001**	**0.97 (0.95; 0.98)**	**<0.001**	**0.97 (0.95; 0.99)**	**<0.001**	**0.97 (0.95; 0.99)**	**<0.001**
Population structure												
Females (%)	1.01 (0.96; 1.05)	0.774			0.99 (0.94; 1.05)	0.721	0.96 (0.90; 1.02)	0.189	0.92 (0.83; 1.01)	0.069	0.91 (0.83; 1.00)	0.054
Mean age (years)	**1.14 (1.06; 1.23)**	**<0.001**			**1.11 (1.03; 1.20)**	**0.009**	1.04 (0.94; 1.14)	0.466	0.91 (0.81; 1.02)	0.095	0.92 (0.81; 1.04)	0.185
Education and income												
Low education (%)	1.06 (0.99; 1.13)	0.102					1.01 (0.95; 1.09)	0.701	1.07 (0.99; 1.16)	0.055	1.07 (0.97; 1.19)	0.178
Unemployment (%)	**1.07 (1.00; 1.14)**	**0.039**					1.02 (0.94; 1.11)	0.661	1.00 (0.90; 1.11)	0.943	1.00 (0.89; 1.11)	0.938
Median annual income (eur)	**0.91 (0.86; 0.96)**	**<0.001**					**0.91 (0.83; 0.99)**	**0.037**	0.96 (0.85; 1.08)	0.496	0.95 (0.83; 1.08)	0.429
Location and housing												
Population density (per km^2)	1.00 (0.99; 1.01)	0.799	**1.02 (1.01; 1.03)**	**<0.001**	**1.02 (1.01; 1.03)**	**0.001**	**1.03 (1.02; 1.04)**	**<0.001**	**1.03 (1.01; 1.06)**	**0.004**	**1.03 (1.01; 1.06)**	**0.007**
Individuals living in rural area (%)	**1.09 (1.04; 1.14)**	**<0.001**							1.05 (0.96; 1.15)	0.287	1.05 (0.96; 1.16)	0.280
Individuals living in municipality of birth (%)	**1.11 (1.06; 1.16)**	**<0.001**							1.00 (0.93; 1.07)	0.926	1.00 (0.93; 1.07)	0.978
Individuals per household unit	**0.92 (0.88; 0.97)**	**0.002**							0.90 (0.79; 1.03)	0.111	0.91 (0.79; 1.05)	0.179
Overcrowded household units (%)	**0.93 (0.87; 0.99)**	**0.028**							0.94 (0.84; 1.05)	0.248	0.94 (0.84; 1.05)	0.244
Other indicators												
Foreign language speakers (%)	0.98 (0.95; 1.01)	0.172	**0.95 (0.92; 0.97)**	**<0.001**	**0.96 (0.94; 0.99)**	**0.007**	0.98 (0.95; 1.00)	0.096	**0.96 (0.93; 0.99)**	**0.009**	**0.96 (0.93; 0.99)**	**0.019**
Divorce rate (per 1000 individuals)	0.94 (0.87; 1.01)	0.102									1.02 (0.92; 1.13)	0.670
Car ownership rate (per individual)	1.02 (0.98; 1.08)	0.329									0.99 (0.89; 1.09)	0.789
Crime rate (per individual)	1.04 (0.96; 1.13)	0.326									1.01 (0.94; 1.09)	0.785

Effect sizes of each predictor are interpreted in standard deviation units.

CI = Confidence interval, P = P value, RR = Rate ratio.
